# Meta‐analysis of postoperative pain using non‐sutured or sutured single‐layer open mesh repair for inguinal hernia

**DOI:** 10.1002/bjs5.50139

**Published:** 2019-02-27

**Authors:** S. van Steensel, L. K. van Vugt, A. K. Al Omar, E. H. H. Mommers, S. O. Breukink, L. P. S. Stassen, B. Winkens, N. D. Bouvy

**Affiliations:** ^1^ Department of Surgery, Maastricht University Medical Centre Maastricht the Netherlands; ^2^ Department of Methodology and Statistics, Maastricht University Medical Centre Maastricht the Netherlands; ^3^ NUTRIM School of Nutrition and Translational Research in Metabolism Maastricht University Maastricht the Netherlands; ^4^ CAPHRI School of Care and Public Health Research Institute Maastricht University Maastricht the Netherlands

## Abstract

**Background:**

Chronic postoperative pain occurs in up to 21·7 per cent of patients undergoing open inguinal hernia repair. Several mesh fixation techniques using glue or self‐gripping meshes have been developed to reduce postoperative pain. The aim of this meta‐analysis was to evaluate RCTs comparing adhesional/self‐gripping and sutured single‐layer open mesh fixations in the repair of inguinal herniation, with postoperative pain as endpoint.

**Methods:**

PubMed, Embase and Cochrane CENTRAL databases were searched systematically for RCTs according to the PRISMA guidelines; the study was registered at PROSPERO (CRD42017056373). Different fixation methods were analysed. The primary outcome, chronic pain, was defined as a postoperative visual analogue scale (VAS) score of at least 3 at 12 months. Secondary outcomes were mean VAS score at 1 week and at 1 month after surgery.

**Results:**

Twenty‐three studies including 5190 patients were included in the meta‐analysis. Adhesional (self‐adhering or glued) or self‐gripping fixation methods were associated with a significantly lower VAS score at 1 week (mean difference –0·49, 95 per cent c.i. ‐0·81 to –0·17; *P = *0·003) and at 1 month (mean difference –0·31, –0·58 to –0·04; *P = *0·02) after surgery than suture fixation, but the incidence of chronic pain after 12 months was similar in the two groups (odds ratio 0·70, 95 per cent c.i. 0·30 to 1·66). Differences in recurrences and complications between groups did not reach statistical significance.

**Conclusion:**

There was no difference in the incidence of chronic pain 12 months after different mesh repair fixation techniques despite significant reductions in short‐term postoperative pain favouring a non‐sutured technique. There were no differences in recurrence rates or in rates of other complications at 1 year.

## Introduction

Inguinal herniation is a common problem, with an estimated lifetime risk of 27 per cent in men and 3 per cent in women^1^, ^2^. Some 20 million people undergo surgical repair each year worldwide^3^. Mesh reinforcement is widely regarded as the standard repair technique based on lower recurrence rates compared with those of primary suture closure^4^, ^5^. Guidelines from the European Hernia Society^6^ recommended two techniques: an open procedure and a laparoscopic totally extraperitoneal (TEP) repair. TEP is not recommended for patients with previous major abdominal surgery, large scrotal hernias, irreducible hernias or recurrences after a posterior approach^6^.

Among complications of inguinal hernia repair, chronic pain is thought to affect 10–21·7 per cent of patients, limiting daily activities in up to one‐quarter of these patients^7^, ^8^, ^9^, ^10^. The incidence and severity of postoperative pain after inguinal hernia repair have been reported, with wide variations largely reflecting lack of standard definitions.

Postherniorrhaphy pain syndrome has been attributed to a variety of causes including neuropathic pain, non‐neuropathic pain (periosteitis of the pubic tubercle, recurrence, adductor tendinitis, ileopectineal bursitis, osteoarthritis) and diffuse tenderness of the spermatic cord^11^.

The incidence of postoperative chronic pain seems higher after open hernia repair compared with a laparoscopic technique^12^. Age also appears to be a factor^13^, as younger patients report pain and functional impairment 1 year after surgery more frequently than those older than 65 years^5^. Patients with high preoperative pain scores also have an increased risk of developing chronic pain^14^.

It has been hypothesized that suture fixation increases the risk of nerve entrapment, causing postoperative pain syndromes^15^. This has led to the development of different mesh fixation products (self‐gripping mesh, mesh with human fibrin glue fixation and cyanoacrylate glue fixation)^16^, ^17^. Although earlier meta‐analyses^18^, ^19^, ^20^, ^21^ considered glue fixation and self‐gripping meshes, none included all self‐adhering or self‐gripping fixation methods. The aim of this meta‐analysis was to evaluate RCTs comparing adhering (glue or self‐adherent) or self‐gripping *versus* sutured mesh repairs, with the endpoints of chronic (primary outcome) and acute (secondary outcome) postoperative pain after a single‐layer mesh repair technique.

## Methods

This meta‐analysis was performed according to the PRISMA guidelines^22^ and registered at PROSPERO (CRD42017056373; http://www.crd.york.ac.uk/PROSPERO/display_record.php?ID=CRD42017056373).

### Eligibility criteria

All RCTs comparing a flat sutured mesh *versus* glue or self‐gripping mesh fixation in inguinal hernia repairs in an adult population with primary, unilateral inguinal hernias were eligible for inclusion. Other study designs (non‐randomized trials, case series) were excluded. Exclusion criteria were mesh plugs and bilayer systems, and methods without fixation or with staples or tacks. Men and women were included. No limitations based on the type of sutures were made, but details were recorded. All studies reporting on postoperative pain were eligible, without limitations based on the definition of postoperative pain. No language restrictions were applied. Unpublished studies were eligible for inclusion.

### Literature search

PubMed, Embase and Cochrane CENTRAL databases were searched systematically on 1 May 2017, using free‐text and Medical Subject Heading (MeSH) terms regarding the target condition (inguinal hernia, groin hernia, ‘Hernia, Inguinal’[MeSH]), operative technique (open repair, mesh repair, Lichtenstein, ‘Surgical Mesh’[MeSH]) and primary outcome (pain, ‘Pain, Postoperative’[MeSH]). The detailed search strategy was made publicly available on PROSPERO (*Table* *S1*, supporting information). The search was designed with the help of an experienced librarian. Reference lists of included articles were searched to identify additional relevant publications.

### Study selection

Results from the database searches were managed using citation manager software (EndNote™ X7; Clarivate Analytics, Philadelphia, Pennsylvania, USA). After removal of duplicates, title and abstract screening and full‐text eligibility assessment was undertaken by two independent authors. Disagreement between the reviewers was discussed. If consensus could not be reached, a third author was contacted for arbitration.

### Data extraction and outcomes

Data extraction was standardized using an electronic data extraction form; the following variables were extracted: trial characteristics (first author, year, sample size, follow‐up), patient characteristics (age, sex, mean BMI, preoperative pain, type of hernia), operative characteristics (experience of the surgeon, type of mesh used, glue type, type of self‐gripping mesh, suture type, neurectomies, pain block) and outcomes (early postoperative pain, chronic postoperative pain, recurrences and procedure time). Chronic postoperative pain was defined as pain scored as 3 or more on a visual or numerical analogue scale (VAS) of 10 cm at 12 months after surgery. Authors were contacted if there were missing data, when additional data were required, or if the reported definition deviated from that mentioned previously. If the corresponding author of the original article did not respond after three reminders, data were excluded. During data extraction, if studies were found to report on the same population, only the relevant, original data were extracted to prevent duplicate inclusion^23^. Data from multiple reports on the same population were combined and considered as a single trial.

### Quality assessment of included studies

Quality assessment was performed by two independent reviewers using the Cochrane risk‐of‐bias tool^24^. This considers random sequence generation, allocation concealment, blinding of participants and personnel, blinding of outcome measurements, completeness of outcome data, outcome reporting and independency of funding in assessment of methodological quality. No studies were excluded from the meta‐analysis based on study quality.

### Data synthesis and statistical analysis

Meta‐analysis was performed using RevMan software version 5.3 (Cochrane Collaboration, Nordic Cochrane Centre, Copenhagen, Denmark). VAS scales were converted into scales from 0 to 10 if needed. Treatment effects on binary outcomes were expressed as pooled odds ratios (ORs) with 95 per cent confidence intervals, calculated using the Mantel–Haenszel method. Differences in numerical variables were expressed as mean differences with 95 per cent confidence intervals and pooled using the inverse‐variance method. A random‐effects model was applied and heterogeneity was expressed using the *I* ^2^ statistic. In addition, 95 per cent prediction interval (PI) values were included.

Subgroup analyses of self‐gripping meshes *versus* glue were undertaken, as well as subgroup analyses based on the type of glue (fibrin *versus* cyanoacrylate) and mesh weight (heavyweight *versus* medium weight *versus* lightweight). A subgroup analysis based on study quality was planned if enough eligible studies with acceptable heterogeneity were available. A funnel plot was prepared to check for publication bias and Egger's regression performed to check for asymmetry.

## Results

**Figure 1:**
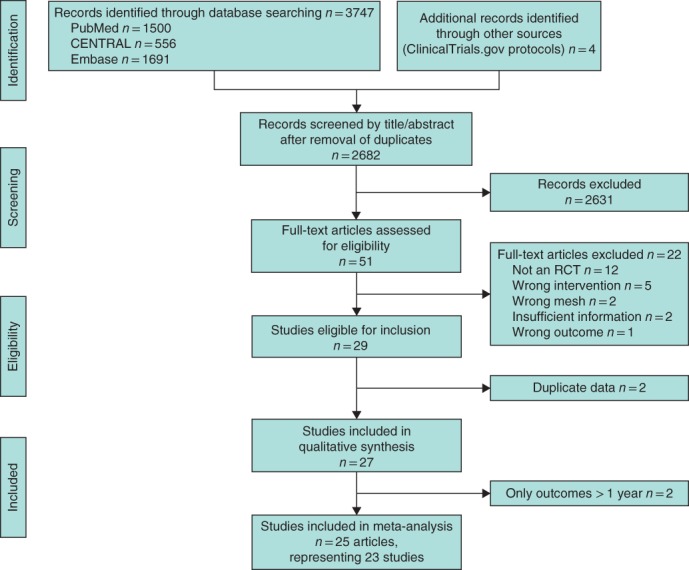
PRISMA diagram showing selection of articles for review

Of 2682 articles identified by the literature search, 51 were selected for full‐text assessment and 29 articles met the inclusion criteria. After removing duplicate articles, 25^16^, ^17^, ^25^, ^26^, ^27^, ^28^, ^29^, ^30^, ^31^, ^32^, ^33^, ^34^, ^35^, ^36^, ^37^, ^38^, ^39^, ^40^, ^41^, ^42^, ^43^, ^44^, ^45^, ^46^, ^47^ were included in the meta‐analysis, representing 23 studies (*Fig*. 1). The study population included in the meta‐analysis consisted of 5190 patients.

Fibrin glue, cyanoacrylate and ProGrip™ self‐gripping mesh (Medtronic, Minneapolis, Minnesota, USA) were the three methods of self‐adhering or self‐gripping fixation encountered in the included studies. One study^45^ had a multiarm trial design comparing cyanoacrylate glue fixation and ProGrip™ self‐gripping mesh with suture fixation of the mesh. Five^16^, ^25^, ^30^, ^32^, ^36^ studies reported on fibrin glue fixation, eight^29^, ^33^, ^37^, ^39^, ^42^, ^43^, ^45^, ^46^ on cyanoacrylate glue fixation and 11^17^, ^26^, ^28^, ^31^, ^34^, ^35^, ^38^, ^40^, ^44^, ^45^, ^47^ on the use of ProGrip™ self‐gripping mesh. Of the 23 articles included in the meta‐analysis, 15^16^, ^17^, ^25^, ^26^, ^28^, ^30^, ^32^, ^33^, ^34^, ^38^, ^39^, ^43^, ^45^, ^46^, ^47^ provided the incidence of chronic pain at 12 months, according to the definition provided in the methods. In two studies^32^, ^33^, data could be extracted directly from the manuscript. Median follow‐up was 12 (range 3–72) months. Study characteristics are summarized in *Table* 1.

**Table 1:** Characteristics of the included studies

Reference	Sample size (men)	Age (years)*	BMI(kg/m^2^)*	Intervention	Mesh (class)	Pain tool	Additional suture fixation	Pain block
Bracale *et al.* 25	52 (49)	56 (46–67)†	25·9 (23·7–27·8)†	Suture	Ultrapro® (I)	VAS 0–10	–	No
	50 (48)	59 (50–67)†	26·0 (24·2–27·3)†	Quixil® (fibrin glue)	Ultrapro® (I)	VAS 0–10	No	No
Bruna Esteban *et al.* 26, 27	45 (38)	49 (19–83)‡	n.a.	Suture	Microval® (I)	VAS 0–10	–	No
	45 (41)	60 (26–80)‡	n.a.	Parietene™ ProGrip™	Parietene™ ProGrip™ (III)	VAS 0–10	No	No
Campanelli *et al.* 16	160 (160)	59 (48–66)†	25·5(2·6)	Suture	Polypropylene macroporous heavyweight (n.a.)	VAS 0–100	No	Yes
	159 (159)	58 (46–65)†	25·5(2·9)	Tissucol® (fibrin glue)	Polypropylene macroporous heavyweight (n.a.)	VAS 0–100	No	Yes
Chatzimavroudis *et al.* 28	25 (23)	62(16)	28·8(3·1)	Suture	Prolene® (II)	VAS 0–10	–	No
	25 (25)	57(18)	27·5(2·9)	Parietene™ ProGrip™	Parietene™ ProGrip™ (III)	VAS 0–10	Yes, non‐absorbable	No
Dąbrowiecki *et al.* 29	21 (21)	45(15)	n.a.	Suture	Prolene® (II)	VAS 0–10	–	n.a.
	20 (20)	47(13)	n.a.	Glubran® sealant (*n*‐butyl‐2‐cyanoacrylate)	Prolene® (II)	VAS 0–10	No	n.a.
Damiano *et al.* 30	252 (238)	55(5)	n.a.	Suture	n.a.	NRS 0–10	–	No
	216 (206)	53(5)	n.a.	Tissucol® (fibrin glue)	n.a.	NRS 0–10	No	No
Fan *et al.* 31	23 (22)	63(5)	n.a.	Suture	Surgipro™ (II)	VAS 0–10	–	n.a.
	22 (18)	62(16)	n.a.	Parietex™ ProGrip™	Parietex™ ProGrip™ (III)	VAS 0–10	Yes, non‐absorbable	n.a.
Fortelny *et al.* 32	20 (18)	54(17)	25·9(3·2)	Suture	Infinit® (II)	VAS 0–100	–	Yes
	18 (16)	47(15)	25·1(4·1)	Tissucol® (fibrin glue)	Infinit® (II)	VAS 0–100	No	Yes
Hoyuela *et al.* 33	182 (162)	59(14)	26·0(3·5)	Suture	Optilene® (I)	VAS 0–10	–	Yes
	188 (170)	61(15)	25·7(3·6)	Histoacryl® (*n*‐butyl‐2‐cyanoacrylate)	Optilene® (I)	VAS 0–10	No	Yes
Jorgensen *et al.* 34	171 (171)	60 (46–68)†	24·8 (23·1–26·7)†	Suture	Parietene Light® (I)	VAS 0–100	–	Yes
	163 (163)	57 (40–65)†	25·2 (23·5–27·1)†	Parietene™ ProGrip™	Parietene™ ProGrip™ (III)	VAS 0–100	No	Yes
Kapischke *et al.* 35	26 (23)	67(12)	n.a.	Suture	Optilene® (I)	VAS 0–100	–	n.a.
	24 (22)	64(13)	n.a.	Parietene™ ProGrip™	Parietene™ ProGrip™ (III)	VAS 0–100	No	n.a.
Karigoudar *et al.* 36	32 (n.a.)	44§	n.a.	Suture	n.a.	VAS 0–100	n.a.	n.a.
	32 (n.a.)	44§	n.a.	Fibrin glue	n.a.	VAS 0–100	n.a.	n.a.
Kim‐Fuchs *et al.* 37	133 (133)	57 (25–83)‡	n.a.	Suture	Vypro® II (III)	n.a.	–	n.a.
	131 (131)	55 (28–85)‡	n.a.	Histoacryl® (*n*‐butyl‐2‐cyanoacrylate)	Vypro® II (III)	n.a.	No	n.a.
Molegraaf *et al.* 38	170 (170)	61(16)	25·0(3·7)	Suture	Parietex™ (I)	VRS 1–6 VAS 0–100	Yes, in 28 patients	Yes
	169 (169)	63(15)	24·9(3·4)	Parietex™ ProGrip™	Parietex™ ProGrip™ (III)	VRS 1–6 VAS 0–100		Yes
Moreno‐Egea^39^	52 (37)	55(14)	29·8(4·2)	Suture	TiMESH® (I)	VAS 0–10	–	No
	50 (34)	57(16)	29·3(3·7)	IFABond™ (*n*‐hexyl‐α‐cyanoacrylate)	TiMESH® (I)	VAS 0–10	No	No
Nikkolo *et al.* 40, 41	75 (68)	54(7)	25·1 (16·6–34·7)‡	Suture	Optilene LP (I)	VAS 0–100	–	n.a.
	70 (65)	58(17)	25·0 (17·4–38·1)‡	Parietex™ ProGrip™	Parietex™ ProGrip™ (III)	VAS 0–100	No	n.a.
Nowobilski *et al.* 42	24 (24)	52 (20–78)‡	n.a.	Suture	n.a.	VAS 0–100	–	Yes
	22 (22)	60 (30–76)‡	n.a.	Indermil™ (butyl‐2‐cyanoacrylate)	n.a.	VAS 0–100	No	Yes
Paajaanen *et al.* 43	151 (135)	53(15)	25(3)	Suture	Optilene® (I)	VAS 0–10, NRS 0–10	–	Yes
	151 (131)	53(15)	25(3)	Glubran® (butyl‐2‐cyanoacrylate)	Optilene® (I)	VAS 0–10, NRS 0–10	No	Yes
Pierides *et al.* 44	196 (182)	53(19–80)‡	25·0 (18·0–33·0)‡	Suture	Parietene Light® (I)	VAS 0–10	–	No
	198 (188)	55 (20–79)‡	24·9 (18·2–36·0)‡	Parietene™ ProGrip™	Parietene™ ProGrip™ (III)	VAS 0–10	No	No
Rönkä *et al.* 45	197 (188)	57(14)	25(3)	Suture	Ultrapro® (I)	VAS 0–10	–	Yes
	211 (194)	59(14)	25(3)	Histoacryl® (butyl‐2‐cyanoacrylate)	Optilene® (I)	VAS 0–10	No	Yes
	189 (182)	56(14)	25(3)	Parietex ProGrip™	Parietex ProGrip™ (III)	VAS 0–10	No	Yes
Sanders *et al.* 17	287 (287)	57(11)	25·5(2·9)	Suture	Parietene Light® (I)	VAS 0–150, SPS 0–150	–	Yes
	270 (270)	57(12)	25·4(3·0)	Parietex ™ ProGrip™	Parietex™ ProGrip™ (III)	VAS 0–150, SPS 0–150	Yes	Yes
Shen *et al.* 46	55 (47)	60(12)	25(2)	Suture	ProLite Ultra™ (II)	VAS 0–10	–	Yes
	55 (45)	63(10)	25(2)	COMPONT® medical adhesive (*n*‐butyl‐2‐cyanoacrylate)	ProLite Ultra™ (II)	VAS 0–10	No	Yes
Verhagen *et al.* 47	181 (179)	58 (19–86)‡	25 (19–36)‡	Suture	Standard polypropylene (n.a.)	VRS 1–6 VAS 0–150	–	n.a.
	182 (175)	60 (20–88)‡	25 (18–33)‡	Parietene™ ProGrip™	Parietene™ ProGrip™ (III)	VRS 1–6 VAS 0–150	No	n.a.

* Values are mean(s.d.) unless indicated otherwise; values are

† median (i.q.r.),

‡ median (range) and §mean. VAS, visual analogue scale; n.a., not available; NRS, numerical rating scale; VRS, visual rating scale; SPS, surgical pain scale. Mesh classification according to Klinge *et al.* 48: class I, large‐pore meshes (textile porosity of 60 per cent or more, or an effective porosity of over 0 per cent); class II, small‐pore meshes (textile porosity of below 60 per cent and without any effective porosity); class III, meshes with special features; class IV, meshes with films; class V, three‐dimensional meshes; class VI, biologicals. Ultrapro®, Prolene®, Vypro® (Ethicon Products, Johnson & Johnson, Somerville, New Jersey, USA); Quixil® (Omrix Biopharmaceuticals, Zaventem, Belgium); Microval® (Microval, Saint‐Just‐Malmont, France); Parietene™ ProGrip™, Surgipro™, Parietex™ ProGrip™, Parietene Light® (Medtronic, Minneapolis, Minnesota, USA); Tissucol® (Baxter Healthcare, Deerfield, Illinois, USA); Glubran® (GEM, Viareggio, Italy); Infinit® (W. L. Gore & Associates, Newark, Delaware, USA); Histoacryl®, Optilene® (Braun, Melsungen, Germany); IFABond™ (Fimed, Domalain, France); TiMESH® (pfm medical UK, Stockport, UK); Indermil™ (Tyco Healthcare Group, Norwalk, Connecticut, USA); COMPONT® (Beijing Compont Medical Devices Beijing, China); ProLite Ultra™ (Atrium Medical, Hudson, New Hampshire, USA).

**Figure 2:**
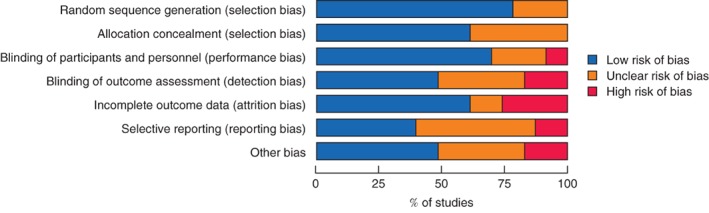
Summary of risk of bias across included studies

**Figure 3:**
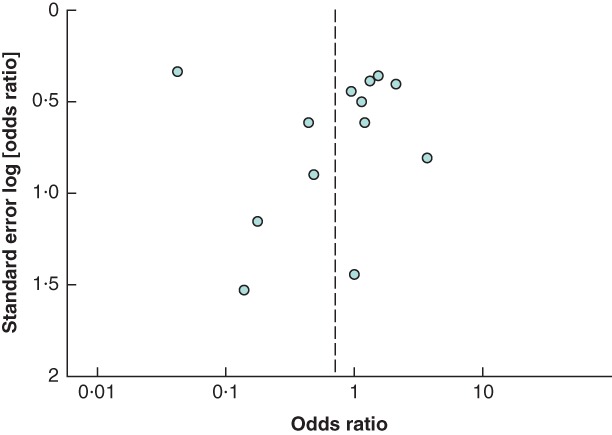
Funnel plot for primary outcome

Four studies^34^, ^38^, ^43^, ^45^ were rated as having a low risk of bias (*Fig*. 2, *Table* 2). Two studies were unblinded^28^, ^37^ and two were single‐blinded^17^, ^25^. The risk of bias was estimated to be high in six studies based on incomplete outcome data^16^, ^17^, ^29^, ^31^, ^33^, ^37^, in two studies^33^, ^37^ based on loss to follow‐up and in two others^17^, ^47^ based on missing VAS scores. Two RCTs were concluded prematurely, one^31^ because of significant results at the interim analysis^32^ and one because of discontinuation of mesh production. Four studies^16^, ^17^, ^44^, ^47^ had a high risk of bias owing to the source of funding. A funnel plot for the primary outcome documented some asymmetry, owing to a lack of studies favouring conventional fixation. Egger's regression showed that the asymmetry was not significant (*P = *0·756) (*Fig*. 3).

**Table 2:** Risk of bias in individual studies


	Random sequence generation (selection bias)	Allocation concealment (selection bias)	Blinding of participants and personnel (performance bias)	Blinding of outcome assessors (detection bias)	Incomplete outcome data (attrition bias)	Selective reporting (reporting bias)	Other bias
Bracale *et al.* 25	+	+	+	–	+	+	+
Bruna Esteban *et al.* 26, 27	+	?	?	?	+	?	+
Campanelli *et al.* 16	+	+	+	+	–	+	–
Chatzimavroudis *et al.* 28	?	?	–	–	+	+	+
Dąbrowiecki *et al.* 29	+	+	+	+	–	?	?
Damiano *et al.* 30	?	?	?	?	?	?	?
Fan *et al.* 31	+	?	+	+	–	+	?
Fortelny *et al.* 32	+	+	?	?	?	?	?
Hoyuela *et al.* 33	+	+	+	+	–	?	+
Jorgensen *et al.* 34	+	+	+	+	+	+	+
Kapischke *et al.* 35	+	?	+	+	+	?	?
Karigoudar *et al.* 36	?	?	?	?	+	–	+
Kim‐Fuchs *et al.* 37	+	+	–	–	–	?	?
Molegraaf *et al.* 38	+	+	+	+	+	+	+
Moreno‐Egea^39^	+	+	+	?	+	?	+
Nikkolo *et al.* 40	+	+	+	?	+	?	+
Nowobilski *et al.* 42	?	?	?	?	+	?	?
Paajanen *et al.* 43	?	+	+	+	+	+	+
Pierides *et al.* 44	+	+	+	+	?	+	–
Rönkä *et al.* 45	+	+	+	+	+	+	+
Sanders *et al.* 17	+	?	+	–	–	–	–
Shen *et al.* 46	+	?	+	?	+	?	?
Verhagen *et al.* 47	+	+	+	+	+	–	–

+, Low risk of bias; –, high risk of bias;?, unclear risk of bias.

**Figure 4:**
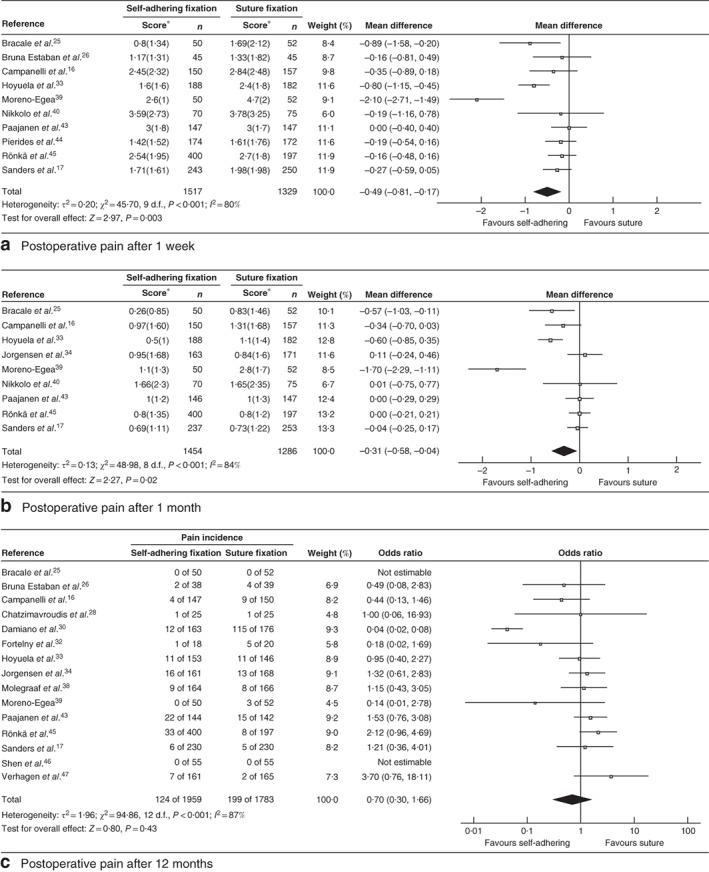
Forest plot comparing postoperative pain after hernia repair with adhesional or self‐gripping fixation *versus* suture fixation. **a** Pain scores after 1 week, **b** pain scores after 1 month and **c** incidence of pain after 12 months. Mean differences and odds ratios are shown with 95 per cent confidence intervals. An inverse‐variance (**a**,**b**) or Mantel–Haenszel (**c**) random‐effects model was used for meta‐analysis. *Values are mean(s.d.)

Ten studies including 2846 participants provided pain data at 1 week, nine studies with 2740 participants at 1 month, and 15 studies with 3742 participants at 12 months (*Fig*. 4 *)*.

Adhering or self‐gripping fixation methods were associated with significantly lower mean VAS scores at 1 week compared with any kind of suture fixation (mean difference –0·49, 95 per cent c.i. –0·81 to –0·17; *P = *0·003; *I* ^2^ = 80 per cent; 95 per cent PI –1·99 to 1·01). After 1 month, the mean VAS score still favoured the non‐sutured group (mean difference –0·31, –0·58 to –0·04; *P = *0·02; *I* ^2^ = 84 per cent; 95 per cent PI –1·70 to 1·08). After 12 months, however, there was no significant difference in the incidence of chronic pain between the two groups (OR 0·70, 95 per cent c.i. 0·30 to 1·66; *P = *0·43; *I* ^2^ = 87 per cent; 95 per cent PI 0·03 to 12·86).

Subgroup analysis showed a significant reduction in mean VAS score for glue fixation *versus* suture fixation after 1 week (mean difference –0·71, –1·22 to –0·20; *P = *0·007; *I* ^2^ = 87 per cent; 95 per cent PI –2·90 to 1·48) and 1 month (mean difference –0·48, –0·86 to –0·11; *P = *0·01; *I* ^2^ = 87 per cent; 95 per cent PI –2·30 to 1·34) of follow‐up (*Figs* *S1* and *S2*, supporting information). In a comparison of glue fixation with suture fixation at 12 months, the difference in the incidence of chronic pain was not significant (OR 0·43, 0·11 to 1·74; *P = *0·24; *I* ^2^ = 92 per cent; 95 per cent PI 0·01 to 21·25) (*Fig*. *S3*, supporting information).

After 12 months, fibrin glue showed a significant reduction in the incidence of chronic pain compared with suture fixation (OR 0·14, 0·02 to 0·78; *P = *0·03, *I* ^2^ = 83 per cent), but the same did not apply to cyanoacrylate glue (OR 1·36, 0·77 to 2·42; *P = *0·29; *I* ^2^ = 29 per cent) (*Fig*. *S3*, supporting information). Cyanoacrylate glue showed a larger decrease in mean VAS score 1 week after surgery (mean difference compared with suture fixation –0·77, –1·48 to –0·05; *P = *0·04; *I* ^2^ = 92 per cent) than fibrin glue (mean difference –0·58, –1·10 to –0·06; *P = *0·03; *I* ^2^ = 31 per cent) (*Fig*. *S1*, supporting information). At 1 month, the decrease in VAS score was comparable between cyanoacrylate and fibrin glue (*Fig*. *S2*, supporting information).

There was no significant difference in VAS score between ProGrip™ and suture fixation at 1 week (mean difference –0·17, –0·35 to 0·02; *P = *0·08; *I* ^2^ = 0 per cent; 95 per cent PI –1·40 to 1·06) and 1 month (–0·00, –0·14 to 0·14; *P = *0·99; *I* ^2^ = 0 per cent; 95 per cent PI –0·41 to 0·41) respectively after operation (*Figs* *S4* and *S5*, supporting information). As regards the incidence of chronic pain at 12 months' follow‐up, there was no significant difference between ProGrip™ and suture fixation (OR 1·45, 0·92 to 2·28; *P = *0·11; *I* ^2^ = 0 per cent; 95 per cent PI 0·19 to 10·73) (*Fig*. *S6*, supporting information).

Subgroup analysis comparing heavyweight and lightweight meshes was abandoned. The small number of articles reporting medium weight or heavyweight meshes was thought to make such an analysis unreliable.

### Recurrence

**Figure 5:**
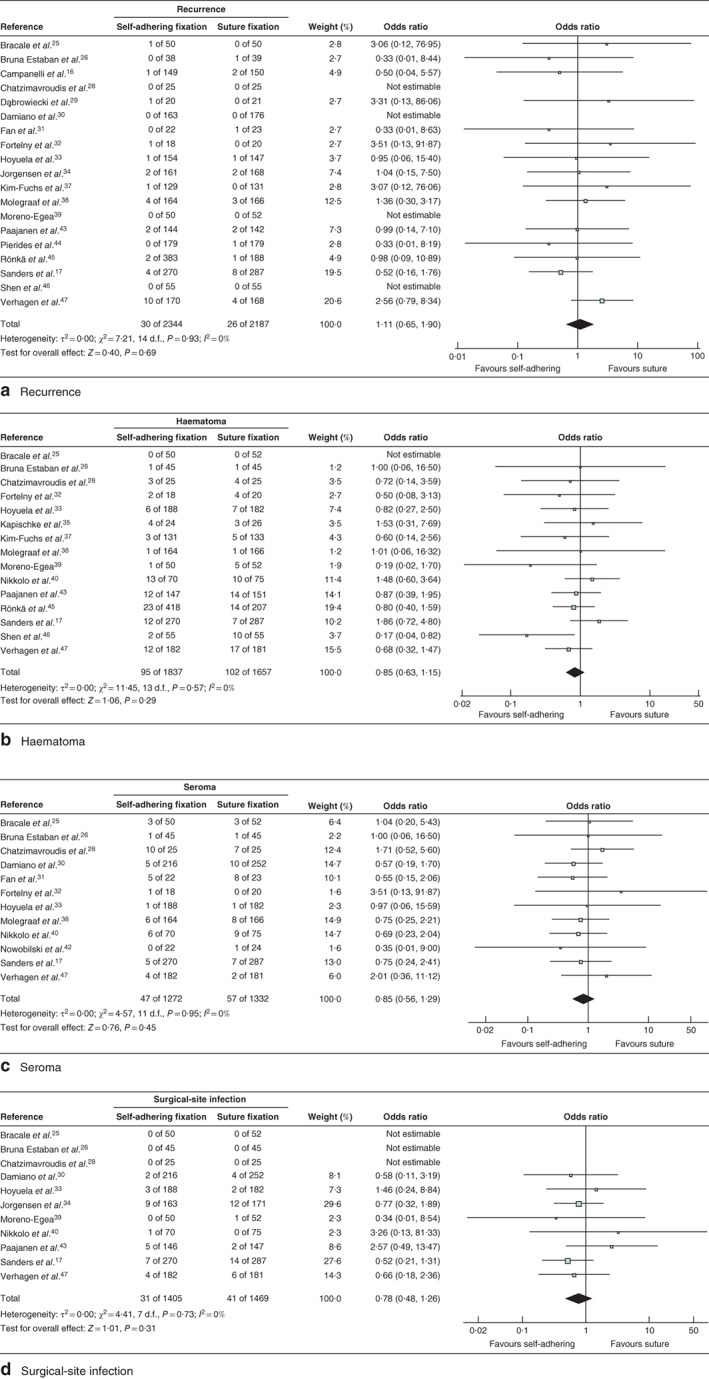
Forest plot comparing rates of recurrence, haematoma, seroma and surgical‐site infection after hernia repair with adhesional or self‐gripping fixation *versus* suture fixation. Rates of **a** recurrence, **b** haematoma, **c** seroma and **d** surgical‐site infection. Odds ratios are shown with 95 per cent confidence intervals. A Mantel–Haenszel random‐effects model was used for meta‐analysis

Nineteen studies^16^, ^17^, ^25^, ^26^, ^28^, ^29^, ^30^, ^31^, ^32^, ^33^, ^34^, ^37^, ^38^, ^39^, ^43^, ^44^, ^45^, ^46^, ^47^ including 4531 patients analysed recurrence. No significant difference was found in recurrence rates between the intervention and control groups after 1 year of follow‐up (OR 1·11, 95 per cent c.i. 0·65 to 1·90; *P = *0·69; *I* ^2^ = 0 per cent; 95 per cent PI 0·15 to 8·23) (*Fig*. 5). In subgroup analyses, ProGrip™ (OR 0·98, 0·52 to 1·86; *P = *0·96; *I* ^2^ = 0 per cent; 95 per cent PI 0·15 to 6·60), fibrin glue (OR 1·34, 0·25 to 7·07; *P = *0·73; *I* ^2^ = 0 per cent; 95 per cent PI not estimable) and cyanoacrylate glue (OR 1·53, 0·48 to 4·86; *P = *0·47; *I* ^2^ = 0 per cent; 95 per cent PI 0·11 to 22·11) showed no significant difference in recurrence rate in separate comparisons *versus* suture fixation.

### Other complications

One study^36^ was excluded as it did not report on complications. Fifteen studies^17^, ^25^, ^26^, ^28^, ^32^, ^33^, ^35^, ^37^, ^38^, ^39^, ^40^, ^43^, ^45^, ^46^, ^47^ reported on haematoma formation, 12^17^, ^25^, ^26^, ^28^, ^30^, ^31^, ^32^, ^33^, ^38^, ^40^, ^42^, ^47^ on seroma formation and 11^17^, ^25^, ^26^, ^28^, ^30^, ^33^, ^34^, ^39^, ^40^, ^43^, ^47^ on surgical‐site infection. Only studies that reported specifically on haematoma, seroma or surgical‐site infection were included in the meta‐analysis. No significant differences were detected between self‐adhering or self‐gripping fixation methods and suture fixation in the occurrence of haematoma (OR 0·85, 95 per cent c.i. 0·63 to 1·15; *P = *0·29; *I* ^2^ = 0 per cent; 95 per cent PI 0·18 to 4·02), seroma (OR 0·85, 0·56 to 1·29; *P = *0·45; *I* ^2^ = 0 per cent; 95 per cent PI 0·19 to 3·76) or surgical‐site infection (OR 0·78, 0·48 to 1·26; *P = *0·31; *I* ^2^ = 0 per cent; 95 per cent PI 0·10 to 6·12 (*Fig*. 5). Furthermore, subgroup analysis for glue fixation or ProGrip™ mesh *versus* suture fixation showed no significant differences in occurrence of haematoma, seroma or surgical‐site infection (*Table* *S2*, supporting information).

### Procedure time

**Figure 6:**
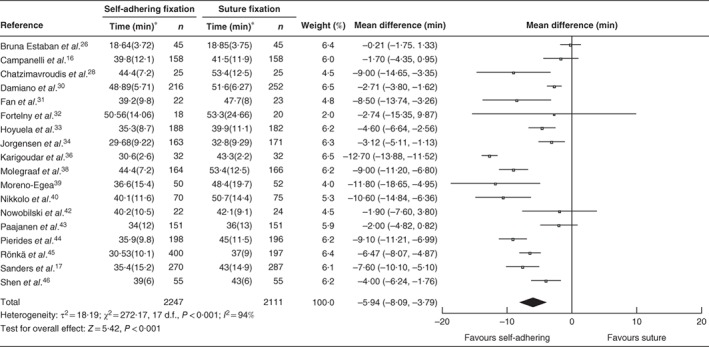
Forest plot comparing procedure times after hernia repair with adhesional or self‐gripping fixation *versus* suture fixation. Mean differences are shown with 95 per cent confidence intervals. An inverse‐variance random‐effects model was used for meta‐analysis. *Values are mean(s.d.)

Eighteen studies reported the duration of operation, including 2247 intervention and 2111 control procedures. There was a significant reduction of 5·94 (95 per cent c.i. –8·09 to −3·79; *P* < 0·001; *I* ^2^ = 94 per cent; 95 per cent PI –14·50 to 2·62) min in procedure time favouring a self‐adhering or self‐gripping mesh fixation over suture fixation (*Fig*. 6).

## Discussion

This meta‐analysis compared RCTs on adhesional and self‐gripping mesh fixation in open inguinal hernia repair, providing significant data regarding the outcome postoperative pain. Discrepancies in the definition of chronic pain between studies were dealt with by directly contacting the authors, asking them to rearrange their data to a preset definition.

Although chronic pain was defined previously as pain persisting 3 months after surgery, most trials reported on chronic pain at 12 months, and this endpoint was chosen as the primary outcome accordingly. On the other hand, a VAS score of 3 was selected arbitrarily as the cut‐off, because this value approaches the tipping point from mild to moderate pain, although variation in cut‐off points of VAS score is evident^49^, ^50^, ^51^.

RCTs included in the analysis were investigated for risk of bias. Even when this was assessed as being relatively low, the non‐significant asymmetry in the funnel plot could be caused by heterogeneity for the primary outcome. Other causes of heterogeneity could be the variety of meshes used in the different studies. Lightweight meshes have been associated with less chronic pain and discomfort, less foreign body sensation and reduced sensory impairment or tenderness compared with heavyweight meshes^52^.

No significant difference in chronic pain at 12 months was found in a comparison of adhesional or self‐gripping methods of fixation *versus* suture fixation. In subgroup analysis, fibrin glue was associated with a significant reduction in the incidence of chronic pain at 12 months. One included study^30^ reported a remarkably high incidence of chronic pain at 12 months' follow‐up in the control group. In this study, only *P* values were mentioned regarding VAS scores at 1‐week and 6‐month follow‐up, so the data were not included in the meta‐analysis. A significant difference was reported at 1 week and 12 months, but not at the 6‐month time point^30^. In the present meta‐analysis, glue fixation of the mesh was associated with a significantly lower VAS score at 1 week and 1 month compared with suture fixation, with pooled mean reduction in postoperative pain score of 0·71 and 0·48 respectively. At 1 week, a greater reduction in VAS score was observed in the cyanoacrylate group in comparison with suture fixation, than for fibrin glue in comparison with suture fixation. The results were largely affected by a single study^39^ with a mean score of 4·7 in the control group, so conclusions should be drawn carefully. The reduction in mean VAS score was comparable at 1 month for cyanoacrylate and fibrin glue (–0·52 and –0·43 respectively).

Optimum VAS cut‐off points after a groin hernia repair were previously defined as a score ranging from 0 to 0·8 (no pain), 0·9–3·2 (mild pain), 3·3–7·1 (moderate pain) and over 7·1 (severe pain) on a 10‐cm scale^49^. Furthermore, a VAS score of 0–3 cm is considered to indicate successful analgesia during treatment^50^. Bearing this in mind, a reduction of 0·71 in a patient group with mild pain already will not constitute a clinically relevant effect and the impact on use of analgesics would be expected to be negligible. Moreover, results of the comparison between glues should be interpreted cautiously and more RCTs are needed to enable definitive conclusions to be drawn.

ProGrip™ self‐gripping mesh provided no benefit compared with suture fixation with respect to postoperative pain at 1 week, 1 month or 12 months after surgery. The resorbable polylactic acid microgrips possibly caused more trauma or induced a less favourable tissue reaction than cyanoacrylate or fibrin glue. Reports included in this meta‐analysis described the use of an occasional additional single suture at the pubic tubercle to facilitate placement and ensure adequate medial overlap, which might also be a cause of pain due to periosteitis of the pubic tubercle^11^ and influence the results.

Subgroup analysis did not detect a difference in recurrence rates for the different adhesional and self‐gripping fixation methods compared with suture fixation. A previous series^53^ documented a high recurrence rate in patients treated with ProGrip™ self‐gripping mesh compared with suture fixation at 3 years of follow‐up, although a high rate of loss to follow‐up affected the analysis. Another study^41^ reported no recurrences in the ProGrip™ self‐gripping group after 3 years, but these results should be further validated. There were no significant differences in rates of other complications such as haematomas, seromas or surgical‐site infection between glue and self‐gripping fixation methods compared with suture fixation. As expected, the procedure time was significantly shorter for non‐sutured methods than for suture fixation although the mean difference was about 6 min, which may not be meaningful.

## Supporting information


**Figure S1**. Forest plot comparing mean VAS‐score of glue‐fixation and suture fixation at 1 week postoperatively.

**Figure S2**. Forest plot comparing mean VAS‐score of glue‐fixation and suture fixation at 1 month postoperatively.

**Figure S3**. Forest plot comparing the incidence of chronic pain (VAS > 3) of glue‐fixation and suture fixation at 12 month postoperatively.

**Figure S4**. Forest plot comparing mean VAS‐score of ProGripÔ and suture fixation at 1 week postoperatively.

**Figure S5**. Forest plot comparing mean VAS‐score of ProGripÔ and suture fixation at 1 month postoperatively.

**Figure S6**. Forest plot comparing the incidence of chronic pain (VAS > 3) of ProGripÔ and suture fixation at 12 months postoperatively.

**Table S1**. Search in Embase, Cochrane CENTRAL, Pubmed

**Table S2**. complications after mesh placement; hematoma, seroma or surgical site infection. Comparing glue fixation to suture fixation and ProGripÔ to suture fixation.
