# Supplementary material for: Meta‐analysis of postoperative pain using non‐sutured or sutured single‐layer open mesh repair for inguinal hernia
Source: BJS Open. 2019 Feb 27;3(3):260–73. doi: 10.1002/bjs5.50139 (PMC6551402; doi:10.1002/bjs5.50139)
Supplement: Supplementary file 1 — Figure S1. Forest plot comparing mean VAS‐score of glue‐fixation and suture fixation at 1 week postoperatively. [file BJS5-3-260-s001.pdf]

Study or Subgroup	Glue fixation Mean	SD	Total	Suture fixation Mean	SD	Total	Weight	Mean Difference IV, Random, 95% CI
**2.1.1 Fibrin glue**								
Bracale 2014	0.8	1.34	50	1.69	2.12	52	14.6%	−0.89 [−1.58, −0.20]
Campanelli 2012	2.487	2.323	150	2.841	2.475	157	16.3%	−0.35 [−0.89, 0.18]
**Subtotal (95% CI)**			**200**			**209**	**30.9%**	**−0.58 [−1.10, −0.06]**
Heterogeneity: $Tau^2 = 0.04$; $Chi^2 = 1.46$, df = 1 (P = 0.23); $I^2 = 31\%$								
Test for overall effect: Z = 2.19 (P = 0.03)								
**2.1.2 Cyanoacrylate glue**								
Hoyuela 2017	1.6	1.6	188	2.4	1.8	182	18.1%	−0.80 [−1.15, −0.45]
Moreno-Egea 2014	2.6	1	50	4.7	2	52	15.5%	−2.10 [−2.71, −1.49]
Paajanen 2011	3	1.8	147	3	1.7	147	17.6%	0.00 [−0.40, 0.40]
Ronka 2015	2.4	2	211	2.7	1.8	197	17.9%	−0.30 [−0.67, 0.07]
**Subtotal (95% CI)**			**596**			**578**	**69.1%**	**−0.77 [−1.48, −0.05]**
Heterogeneity: $Tau^2 = 0.48$; $Chi^2 = 35.65$, df = 3 (P < 0.00001); $I^2 = 92\%$								
Test for overall effect: Z = 2.11 (P = 0.04)								
**Total (95% CI)**			**796**			**787**	**100.0%**	**−0.71 [−1.22, −0.20]**
Heterogeneity: $Tau^2 = 0.35$; $Chi^2 = 37.12$, df = 5 (P < 0.00001); $I^2 = 87\%$								
Test for overall effect: Z = 2.71 (P = 0.007)								
Test for subgroup differences: $Chi^2 = 0.18$, df = 1 (P = 0.67), $I^2 = 0\%$								

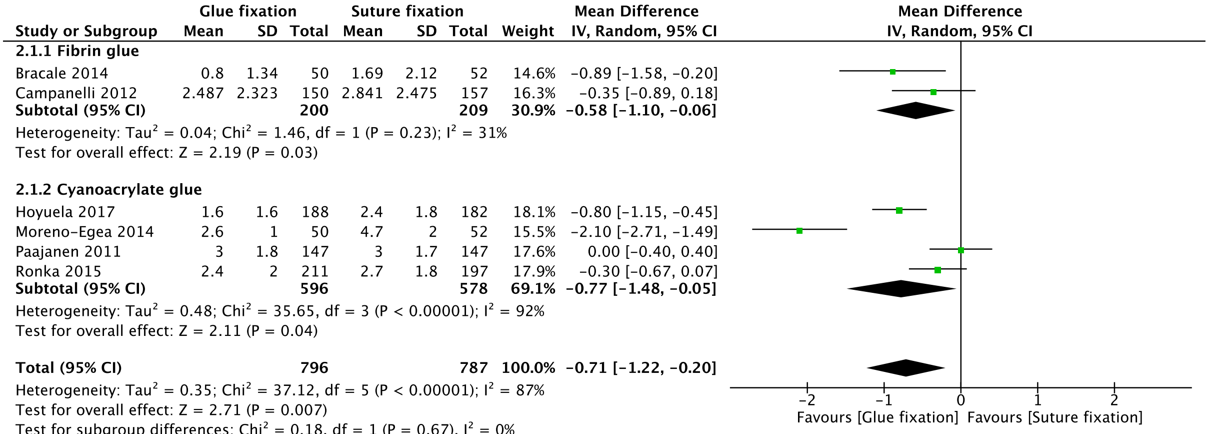

Supporting information: figure 1 Forest plot comparing mean VAS-score of glue-fixation and suture fixation at 1 week postoperatively.
