# Supplementary material for: Meta‐analysis of postoperative pain using non‐sutured or sutured single‐layer open mesh repair for inguinal hernia
Source: BJS Open. 2019 Feb 27;3(3):260–73. doi: 10.1002/bjs5.50139 (PMC6551402; doi:10.1002/bjs5.50139)
Supplement: Supplementary file 2 — Figure S2. Forest plot comparing mean VAS‐score of glue‐fixation and suture fixation at 1 month postoperatively. [file BJS5-3-260-s002.pdf]

Study or Subgroup	Glue fixation Mean	SD	Total	Suture fixation Mean	SD	Total	Weight	Mean Difference IV, Random, 95% CI
**2.2.1 Fibrin glue**								
Bracale 2014	0.26	0.85	50	0.83	1.46	52	15.3%	−0.57 [−1.03, −0.11]
Campanelli 2012	0.971	1.604	150	1.308	1.683	157	16.7%	−0.34 [−0.70, 0.03]
**Subtotal (95% CI)**			**200**			**209**	**32.1%**	**−0.43 [−0.72, −0.14]**
Heterogeneity: Tau² = 0.00; Chi² = 0.60, df = 1 (P = 0.44); I² = 0%								
Test for overall effect: Z = 2.91 (P = 0.004)								
**2.2.2 Cyanoacrylate glue**								
Hoyuela 2017	0.5	1	188	1.1	1.4	182	18.3%	−0.60 [−0.85, −0.35]
Moreno-Egea 2014	1.1	1.3	50	2.8	1.7	52	13.4%	−1.70 [−2.29, −1.11]
Paajanen 2011	1	1.2	146	1	1.3	147	17.9%	0.00 [−0.29, 0.29]
Ronka 2015	0.8	1.4	211	0.8	1.2	197	18.3%	0.00 [−0.25, 0.25]
**Subtotal (95% CI)**			**595**			**578**	**67.9%**	**−0.52 [−1.06, 0.02]**
Heterogeneity: Tau² = 0.27; Chi² = 37.13, df = 3 (P < 0.00001); I² = 92%								
Test for overall effect: Z = 1.88 (P = 0.06)								
**Total (95% CI)**			**795**			**787**	**100.0%**	**−0.48 [−0.86, −0.11]**
Heterogeneity: Tau² = 0.19; Chi² = 38.22, df = 5 (P < 0.00001); I² = 87%								
Test for overall effect: Z = 2.51 (P = 0.01)								
Test for subgroup differences: Chi² = 0.08, df = 1 (P = 0.77), I² = 0%								

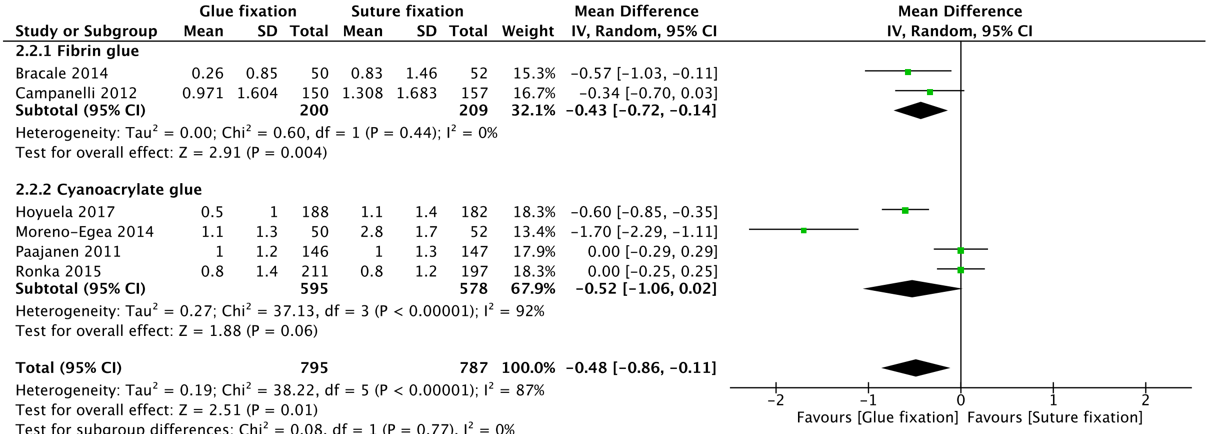

Supporting information: figure 2 Forest plot comparing mean VAS-score of glue-fixation and suture fixation at 1 month postoperatively.
