# Supplementary material for: Meta‐analysis of postoperative pain using non‐sutured or sutured single‐layer open mesh repair for inguinal hernia
Source: BJS Open. 2019 Feb 27;3(3):260–73. doi: 10.1002/bjs5.50139 (PMC6551402; doi:10.1002/bjs5.50139)
Supplement: Supplementary file 3 — Figure S3. Forest plot comparing the incidence of chronic pain (VAS > 3) of glue‐fixation and suture fixation at 12 month postoperatively. [file BJS5-3-260-s003.pdf]

Study or Subgroup	Glue fixation Events	Glue fixation Total	Suture fixation Events	Suture fixation Total	Weight	Odds Ratio M-H, Random, 95% CI
**2.3.1 Fibrin glue**						
Bracale 2014	0	50	0	52		Not estimable
Campanelli 2012	4	147	9	150	14.9%	0.44 [0.13, 1.46]
Damiano 2014	12	163	115	176	16.2%	0.04 [0.02, 0.08]
Fortelny 2014	1	18	5	20	11.7%	0.18 [0.02, 1.69]
**Subtotal (95% CI)**		**378**		**398**	**42.8%**	**0.14 [0.02, 0.78]**
Total events	17		129			
Heterogeneity: $Tau^2 = 1.86$; $Chi^2 = 11.73$, df = 2 (P = 0.003); $I^2 = 83\%$						
Test for overall effect: Z = 2.24 (P = 0.03)						
**2.3.2 Cyanoacrylate glue**						
Hoyuela 2017	11	153	11	146	15.8%	0.95 [0.40, 2.27]
Moreno-Egea 2014	0	50	3	52	9.5%	0.14 [0.01, 2.78]
Paajanen 2011	22	144	15	142	16.1%	1.53 [0.76, 3.08]
Ronka 2015	18	211	8	197	15.8%	2.20 [0.94, 5.19]
Shen 2012	0	55	0	55		Not estimable
**Subtotal (95% CI)**		**613**		**592**	**57.2%**	**1.36 [0.77, 2.42]**
Total events	51		37			
Heterogeneity: $Tau^2 = 0.10$; $Chi^2 = 4.20$, df = 3 (P = 0.24); $I^2 = 29\%$						
Test for overall effect: Z = 1.06 (P = 0.29)						
**Total (95% CI)**		**991**		**990**	**100.0%**	**0.43 [0.11, 1.74]**
Total events	68		166			
Heterogeneity: $Tau^2 = 3.00$; $Chi^2 = 78.76$, df = 6 (P < 0.00001); $I^2 = 92\%$						
Test for overall effect: Z = 1.18 (P = 0.24)						
Test for subgroup differences: $Chi^2 = 6.04$, df = 1 (P = 0.01), $I^2 = 83.4\%$						

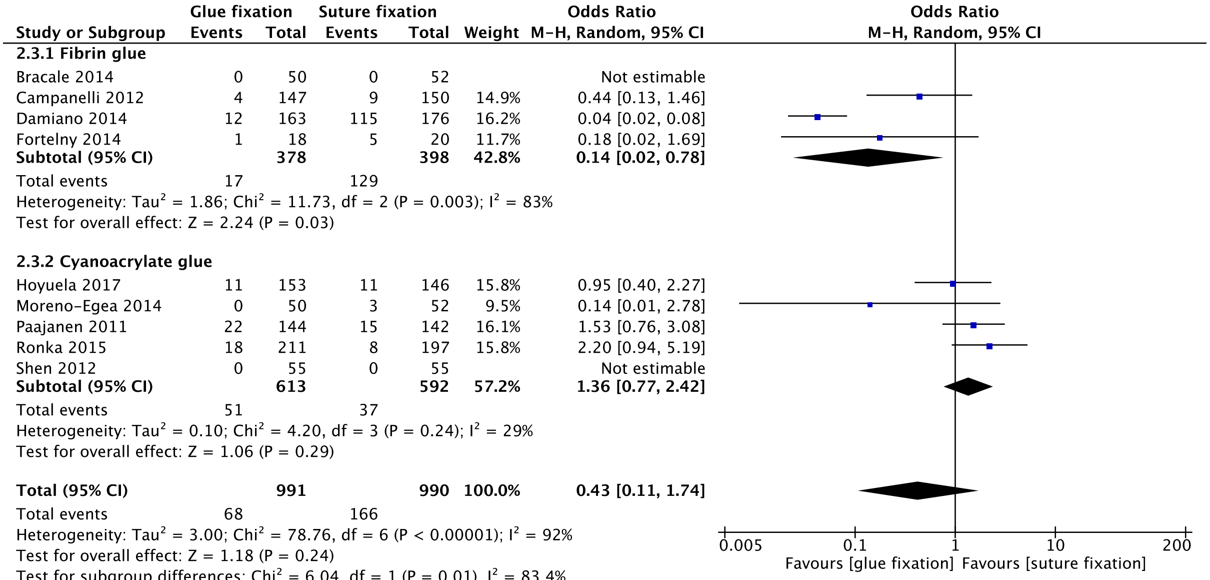

Supporting information: figure 3 Forest plot comparing the incidence of chronic pain (VAS>3) of glue-fixation and suture fixation at 12 month postoperatively.
