# Supplementary material for: Meta‐analysis of postoperative pain using non‐sutured or sutured single‐layer open mesh repair for inguinal hernia
Source: BJS Open. 2019 Feb 27;3(3):260–73. doi: 10.1002/bjs5.50139 (PMC6551402; doi:10.1002/bjs5.50139)
Supplement: Supplementary file 4 — Figure S4. Forest plot comparing mean VAS‐score of ProGripÔ and suture fixation at 1 week postoperatively. [file BJS5-3-260-s004.pdf]

	Progrip			Suture fixation				Mean Difference
**Study or Subgroup**	**Mean**	**SD**	**Total**	**Mean**	**SD**	**Total**	**Weight**	**IV, Random, 95% CI**
Bruna Estaban 2010	1.17	1.305	45	1.33	1.822	45	8.1%	−0.16 [−0.81, 0.49]
Nikkolo 2015	3.59	2.73	70	3.78	3.25	75	3.6%	−0.19 [−1.16, 0.78]
Pierides 2012	1.42	1.52	174	1.61	1.76	172	28.8%	−0.19 [−0.54, 0.16]
Ronka 2015	2.7	1.9	189	2.7	1.8	197	25.3%	0.00 [−0.37, 0.37]
Sanders 2014	1.71	1.61	243	1.98	1.98	250	34.2%	−0.27 [−0.59, 0.05]
**Total (95% CI)**			**721**			**739**	**100.0%**	**−0.17 [−0.35, 0.02]**

Heterogeneity: $Tau^2 = 0.00$; $Chi^2 = 1.21$, df = 4 (P = 0.88); $I^2 = 0\%$
Test for overall effect: Z = 1.76 (P = 0.08)

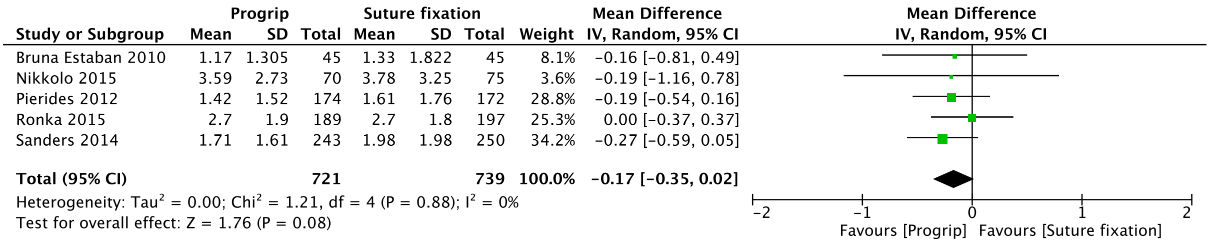

Supporting information: figure 4 Forest plot comparing mean VAS-score of ProGrip™ and suture fixation at 1 week postoperatively.
