# Supplementary material for: Meta‐analysis of postoperative pain using non‐sutured or sutured single‐layer open mesh repair for inguinal hernia
Source: BJS Open. 2019 Feb 27;3(3):260–73. doi: 10.1002/bjs5.50139 (PMC6551402; doi:10.1002/bjs5.50139)
Supplement: Supplementary file 5 — Figure S5. Forest plot comparing mean VAS‐score of ProGripÔ and suture fixation at 1 month postoperatively. [file BJS5-3-260-s005.pdf]

	Progrip			Suture fixation				Mean Difference
Study or Subgroup	Mean	SD	Total	Mean	SD	Total	Weight	IV, Random, 95% CI
Jorgensen 2013	0.95	1.675	163	0.839	1.6	171	16.4%	0.11 [−0.24, 0.46]
Nikkolo 2015	1.66	2.3	70	1.65	2.35	75	3.5%	0.01 [−0.75, 0.77]
Ronka 2015	0.8	1.3	189	0.8	1.2	197	32.5%	0.00 [−0.25, 0.25]
Sanders 2014	0.69	1.11	237	0.73	1.22	253	47.6%	−0.04 [−0.25, 0.17]
**Total (95% CI)**			**659**			**696**	**100.0%**	**−0.00 [−0.14, 0.14]**

Heterogeneity: $Tau^2 = 0.00$; $Chi^2 = 0.53$, df = 3 (P = 0.91); $I^2 = 0\%$
Test for overall effect: Z = 0.01 (P = 0.99)

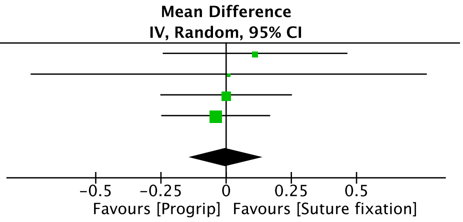

Supporting information: figure 5 Forest plot comparing mean VAS-score of ProGrip™ and suture fixation at 1 month postoperatively.
