# Supplementary material for: Meta‐analysis of postoperative pain using non‐sutured or sutured single‐layer open mesh repair for inguinal hernia
Source: BJS Open. 2019 Feb 27;3(3):260–73. doi: 10.1002/bjs5.50139 (PMC6551402; doi:10.1002/bjs5.50139)
Supplement: Supplementary file 6 — Figure S6. Forest plot comparing the incidence of chronic pain (VAS > 3) of ProGripÔ and suture fixation at 12 months postoperatively. [file BJS5-3-260-s006.pdf]

Study or Subgroup	Progrip Events	Progrip Total	Suture fixation Events	Suture fixation Total	Weight	Odds Ratio M–H, Random, 95% CI
Bruna Estaban 2010	2	38	4	39	6.6%	0.49 [0.08, 2.83]
Chatzimavroudis 2014	1	25	1	25	2.6%	1.00 [0.06, 16.93]
Jorgensen 2013	16	161	13	168	34.9%	1.32 [0.61, 2.83]
Molegraaf 2017	9	164	8	166	21.4%	1.15 [0.43, 3.05]
Ronka 2015	15	189	8	197	26.3%	2.04 [0.84, 4.92]
Verhagen 2016	7	161	2	165	8.1%	3.70 [0.76, 18.11]
**Total (95% CI)**		**738**		**760**	**100.0%**	**1.45 [0.92, 2.28]**
Total events	50		36			

Heterogeneity: Tau^2 = 0.00; Chi^2 = 3.75, df = 5 (P = 0.59); I^2 = 0%

Test for overall effect: Z = 1.61 (P = 0.11)

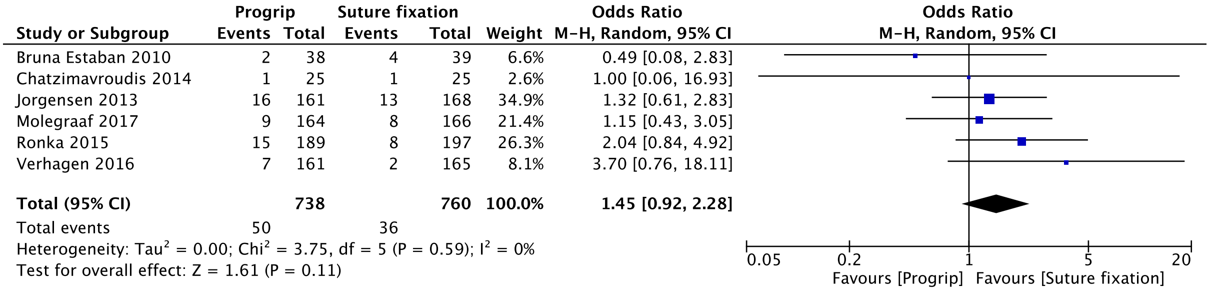

Supporting information: figure 6 Forest plot comparing the incidence of chronic pain (VAS>3) of ProGrip™ and suture fixation at 12 months postoperatively.
