# Supplementary material for: Meta‐analysis of postoperative pain using non‐sutured or sutured single‐layer open mesh repair for inguinal hernia
Source: BJS Open. 2019 Feb 27;3(3):260–73. doi: 10.1002/bjs5.50139 (PMC6551402; doi:10.1002/bjs5.50139)
Supplement: Supplementary file 7 — Table S1. Search in Embase, Cochrane CENTRAL, Pubmed [file BJS5-3-260-s007.pdf]

Supporting information: table 1 Search in Embase, Cochrane CENTRAL, Pubmed

Embase

1. inguinal hernia/
2. hernioplasty/ or surgical mesh/ or lichtenstein.mp.
3. postoperative pain/ or pain/
4. 1 and 2
5. 3 and 4

Cochrane CENTRAL

ID	Search
#1	(inguinal hernia) or groin hernia
#2	MeSH descriptor: [Hernia, Inguinal] explode all trees
#3	(((open repair) or mesh repair) or Lichtenstein)
#4	MeSH descriptor: [Surgical Mesh] explode all trees
#5	pain
#6	MeSH descriptor: [Pain, Postoperative] explode all trees
#7	(#1) or #2
#8	#3 or #4
#9	#5 or #6
#10	((#7) and #8) and #9

Pubmed

(((("hernia, inguinal"[MeSH Terms] OR ("hernia"[All Fields] AND "inguinal"[All Fields]) OR "inguinal hernia"[All Fields] OR ("inguinal"[All Fields] AND "hernia"[All Fields])) OR (("groin"[MeSH Terms] OR "groin"[All Fields]) AND ("hernia"[MeSH Terms] OR "hernia"[All Fields]))) OR "Hernia, Inguinal"[Mesh]) AND ((((open[All Fields] AND "repair"[All Fields]) OR ("mesh"[All Fields] AND "repair"[All Fields])) OR Lichtenstein[All Fields]) OR "Surgical Mesh"[Mesh])) AND (("pain"[MeSH Terms] OR "pain"[All Fields]) OR "Pain, Postoperative"[Mesh])
