# Supplementary material for: Meta‐analysis of postoperative pain using non‐sutured or sutured single‐layer open mesh repair for inguinal hernia
Source: BJS Open. 2019 Feb 27;3(3):260–73. doi: 10.1002/bjs5.50139 (PMC6551402; doi:10.1002/bjs5.50139)
Supplement: Supplementary file 8 — Table S2. complications after mesh placement; hematoma, seroma or surgical site infection. Comparing glue fixation to suture fixation and ProGripÔ to suture fixation. [file BJS5-3-260-s008.pdf]

Table 2 complications after mesh placement; hematoma, seroma or surgical site infection. Comparing glue fixation to suture fixation and ProGrip™ to suture fixation.

	Glue fixation vs Suture fixation<br>OR (95%CI) p-value	ProGrip™ vs Suture fixation<br>OR (95%CI) p-value
Hematoma	0.70 (0.45 to 1.07) p=0.10<br>PI 95% (0.13 to 3.74)	1.00 (0.67 to 1.48) p=0.98<br>PI 95% (0.40 to 2.49)
Seroma	0.75 (0.33 to 1.68) p=0.49<br>PI 95% (0.04 to 15.76)	0.89 (0.55 to 1.45) p=0.64<br>PI 95% (0.25 to 3.23)
Surgical Site infection	1.16 (0.45 to 3.01) p=0.75<br>PI 95% (0.01 to 97.15)	0.67 (0.38 to 1.19) p=0.17<br>PI 95% (0.02 to 29.32)
